# Psychometric study of the European Portuguese version of the PedsQL 3.0 Cancer Module

**DOI:** 10.1186/s12955-016-0421-y

**Published:** 2016-02-13

**Authors:** Susana Santos, Carla Crespo, M. Cristina Canavarro, Ananda Fernandes, Luís Batalha, Diana de Campos, Armando Pinto

**Affiliations:** Faculty of Psychology and Educational Sciences, University of Coimbra, Coimbra, Portugal; Faculdade de Psicologia, Universidade de Lisboa, Lisbon, Portugal; UICISA: E, Escola Superior de Enfermagem de Coimbra, Coimbra, Portugal; Portuguese Institute of Oncology (IPO-Porto), Porto, Portugal

**Keywords:** European Portuguese version, PedsQL™ 3.0 Cancer Module, Health-related quality of life, Pediatric cancer, Psychometric properties

## Abstract

**Background:**

Health-related quality of life (HRQoL) is an important outcome to assess the impact of cancer. This article examines the psychometric properties of the European Portuguese self-report version of the Pediatric Quality of Life Inventory™ Cancer Module (PedsQL™ 3.0 Cancer Module) in children and adolescents with cancer.

**Methods:**

The participants, 332 children/adolescents diagnosed with cancer (8–18 years old), completed measures to assess HRQoL (PedsQL™ 3.0 Cancer Module and DISABKIDS Chronic Generic Measure; DCGM-12) and anxiety (Revised Children’s Manifest Anxiety Scale - second edition; RCMAS-2). A subsample (*n* = 52) completed the PedsQL™ 3.0 Cancer Module a second time following one-week. The pediatric oncologists completed the Intensity of Treatment Rating Scale 3.0 (ITR 3.0).

**Results:**

For the whole sample, the PedsQL™ 3.0 Cancer Module demonstrated good item discrimination (*r*s = .30 to .54). The confirmatory factor analysis testing the presence of eight first-order factors loading significantly in a second-order factor revealed an acceptable fit (CFI = .91; RMSEA = .05). The correlation of PedsQL™ 3.0 Cancer Module with DCGM-12 (*r*s = .17 to .58), and with RCMAS-2 (*r*s = −.16 to–.51) attested convergent validity. This inventory demonstrated minimally acceptable to very good internal consistency (*α*s = .65 to .87) and temporal stability (ICCs = .61 to .81).

**Conclusions:**

These findings demonstrate that the European Portuguese self-report version of the PedsQL™ 3.0 Cancer Module is a valid and reliable instrument for assessing HRQoL in pediatric cancer.

**Electronic supplementary material:**

The online version of this article (doi:10.1186/s12955-016-0421-y) contains supplementary material, which is available to authorized users.

## Background

According to the World Health Organization, the global incidence rate of childhood cancer is approximately 100–150 cases per million children and adolescents under 15 years of age [1]. In Europe, namely in Portugal, as well as in North America, leukemias are the most common childhood cancers, followed by central nervous system tumors and lymphomas [2].

Clinical advancements in the treatment of this condition have led to a relevant reduction in mortality rates in the last 50 years. Currently, 70 to 80 % of pediatric cancer patients can be cured in developed countries [3] if diagnosed and treated early [4]. This progress, however, has been achieved with aggressive medical protocols that have a significant impact on children and adolescents’ daily lives, which affect, among other aspects, their health-related quality of life (HRQoL) [5]. As survivorship rates have increased concerns regarding patients’ HRQoL have become key aspects in cancer care.

HRQoL is a component of the more global construct of quality of life [1] and has been defined as the patient’s perception of the impact of the disease and treatment in several domains, such as physical, mental and social [6]. Although HRQoL is, by definition, self-referential, the assessment of HRQoL in children and adolescents has, until recently, primarily relied on parents’ reports [e.g., 7]. However, recent studies highlight that child reports cannot be substituted with the parent reports, with the exception of patients too young, too cognitively impaired, or too ill or fatigued to complete a HRQoL assessment instrument [8]. Hence, similar to North America, it is important to make available a reliable and valid assessment instrument in Portugal to measure children and adolescents’ self-reported HRQoL in pediatric cancer patients across different statuses of treatment [9].

A recent systematic review [10] identified 13 instruments for assessing quality of life, which were developed for use with children with cancer and childhood cancer survivors. Nonetheless, only two, the Pediatric Quality of Life Inventory™ Cancer Module (PedsQL™ 3.0 Cancer Module) and the Quality of Life for Children with Cancer Scale, were appropriate taking into account the following requirements: (1) self-reported measure; (2) appropriate for any type of cancer; (3) appropriate for both on- and off-treatment status; and (4) comprising versions for both children and adolescents. Nevertheless, none of them had yet been translated and validated for Portuguese. The option for the PedsQL™ 3.0 Cancer Module was based on the fact that it was currently the most widely used measure of child health [11] and had more psychometric data published compared with other pediatric quality of life instruments [10].

However, to our knowledge, no published studies have examined the factorial structure of the PedsQL™ 3.0 Cancer Module using confirmatory factor analysis (CFA). While exploratory factor analysis (EFA) is designed for situations where links between the observed and latent variables are unknown or uncertain, CFA enables a specific hypothesized structure to be tested [12]. Because the PedsQL™ 3.0 Cancer Module was developed based on a theoretically driven model [13], the use of CFA is appropriate.

Regarding the convergent validity of the self-report version, with minor exceptions, previous studies [14–17] have identified significant positive associations between the scores of the PedsQL™ 3.0 Cancer Module and the PedsQL™ 4.0 Generic Core Scales [13].

In addition, previous studies examining the reliability of the PedsQL™ 3.0 Cancer Module self-report version have demonstrated adequate values of internal consistency [16, 17] and temporal stability [14, 15] for the total score. Regarding the subscales, some studies found values below the established cut points for internal consistency [18] and temporal stability [14, 15].

Despite the growing support for the validity and reliability of the PedsQL™ 3.0 Cancer Module, to date, there is no evidence for its factorial validity, via CFA. In addition, the convergent validity and the test-retest reliability across age groups [for two exceptions: 14, 15] remain understudied.

The present research aimed at examining the psychometric properties of the self-reported European Portuguese version of the PedsQL™ 3.0 Cancer Module in children/adolescents. The specific objectives were to: (1) conduct descriptive and item analyses for the whole sample; (2a) test the factorial validity via CFA for the whole sample; (2b) evaluate convergent validity in two age groups (children 8–12 vs. adolescents 13–18); (3) assess the internal consistency, and the test-retest reliability in two age groups (children 8–12 vs. adolescents 13–18).

## Methods

### Participants

The sample was composed of 332 children/adolescents (174 boys and 158 girls) aged 8 to 18 years old (*M* = 13.0; *SD* = 3.2) who were diagnosed with leukemias (*n* = 131, 39.5 %), lymphomas (*n* = 83, 25.0 %), solid tumor – non central nervous system (*n* = 86, 25.9 %), and central nervous system tumors (*n* = 32, 9.6 %). The majority of children/adolescents had not experienced a relapse (*n* = 287, 86.4 %). Most children/adolescents (*n* = 186, 56.0 %) belonged to families with low socioeconomic status.

The participants were divided into two age groups: children (*n* = 143) aged 8 to 12 years old (*M* = 9.8; *SD* = 1.6) and adolescents (*n* = 189) aged 13 to 18 years old (*M* = 15.5; *SD* = 1.5). With regard to the main sociodemographic and clinical characteristics, the groups differed according to sex. Compared with the group of adolescents, the children’s group had a higher percentage of boys. The groups did not differ in socioeconomic status, treatment status, or intensity of treatment. The sociodemographic and clinical data of the sample are shown in Table 1.

**Table 1:** Sociodemographic and Clinical Characteristics of the Sample

	Whole sample		Children		Adolescents		
	*N* = 332		*n* = 143		*n* = 189		
	*n*	%	*n*	%	*n*	%	χ ^2^ Group differences
Sociodemographic							
Sex							χ^2^_(1)_ = 4.50; *p* = .034; Cramér’s V = .12
Male	174	52.4	85	59.4	89	47.1	
Female	158	47.6	58	40.6	100	52.9	
Socioeconomic status							χ^2^_(2)_ = 2.98; *p* = .226; Cramér’s V = .10
Low	186	56.0	75	52.4	111	58.7	
Medium	111	33.4	55	38.5	56	29.6	
High	35	10.5	13	9.1	22	11.6	
Clinical							
Treatment status							χ^2^_(1)_ = 0.85; *p* = .357; Cramér’s V = .06
On-treatment	161	48.5	74	51.7	87	46.0	
Off-treatment	171	51.5	69	48.3	102	54.0	
Intensity of treatment							χ^2^_(3)_ = 4.59; *p* = .205; Cramér’s V = .12
Level 1: Least intensive	12	3.6	2	1.4	10	5.3	
Level 2: Moderately intensive	112	33.7	53	37.1	59	31.2	
Level 3: Very intensive	153	46.1	63	44.1	90	47.6	
Level 4: Most intensive	55	16.6	25	17.5	30	15.9	

### Procedure

The present study was approved by the Ethics Committees of three Portuguese public hospitals: the Portuguese Institute of Oncology and São João Hospital, both located in Porto, and the Pediatric Department, Centro Hospitalar e Universitário de Coimbra in Coimbra. Between June 2012 and September 2013 all participants who met inclusion criteria were invited to participate, using a consecutive sampling approach. Inclusion criteria consisted of having a cancer diagnosis for at least 3 months; aged 8–18 years; receiving treatment for primary diagnosed/relapsed cancer (on-treatment) or had finished antineoplastic treatments for primary diagnosed/relapsed cancer within the last 60 months (off-treatment). The exclusion criteria consisted of comorbidity with other chronic illness (e.g., diabetes); major developmental disorders (e.g., down syndrome); or end-of-life care. Of the 335 participants approached to participate, nearly all (*n* = 332) provided data (99.4 %). The two participants that declined participation indicated that they were too busy or not interested. The study aims were explained to all participants, and informed consents were obtained from all parents and from participants aged 13 years or more; assent was obtained from the younger children in accordance with the Declaration of Helsinki. The protocol was administered in a separate room in either the inpatient (*n* = 35, 10.5 %) or outpatient (*n* = 297, 89.5 %) setting in the presence of a trained undergraduate and a graduate research assistant. The participants who were scheduled to revisit the hospital within a 1-week period were invited to complete the PedsQL™ 3.0 Cancer Module a second time. Twenty-two children (*M* = 9.9; *SD* = 1.5) and thirty adolescents (*M* = 15.1; *SD* = 1.5) on-treatment agreed to do so and were included in the test-retest analyses.

Linguistic validation was conducted for the PedsQL™ 3.0 Cancer Module, following the PedsQL Linguistic Validation Guidelines [19]. The original scale was translated independently by two bilingual translators whose native language was Portuguese. The two translated versions were analyzed by a research group composed of five members (two psychologists and three nurses), who agreed on a single reconciled version. A third bilingual translator conducted back-translation of this reconciled version. The comparison of the back-translation with the original source version found no inaccuracies in the translation. Then, a face-to-face interview was conducted by a graduate research assistant that inquired whether the participants, five children and five adolescents with cancer who were Portuguese native speakers, had any difficulty in understanding the scale’s items; the patients’ interpretations of the items were also examined. Because the participants did not report difficulty in understanding the items or in using the response scale, the reconciled version was adopted as the final version.

### Measures

#### HRQoL (Cancer-Specific)

The PedsQL™ 3.0 Cancer Module [13] is the most updated version of the initial PedsQL™ 1.0 Cancer Module [20]. There is a self-report version for children (age range 8–12 years) and another one for adolescents (age range 13–18 years), that differs only in the wording of the instructions (i.e., “child(ren)” vs. “teen(s)”). Both these versions are composed of 27 items grouped into eight subscales: Pain and Hurt (2 items), Nausea (5 items), Procedural Anxiety (3 items), Treatment Anxiety (3 items), Worry (3 items), Cognitive Problems (5 items), Perceived Physical Appearance (3 items), and Communication (3 items). The participants evaluated how frequently a specific problem occurred in the past one month (e.g., “I worry about side effects from medical treatments”) using a 5-point Likert scale ranging from 0 (*never*) to 4 (*almost always*). For more detail about the Portuguese and English originals versions of the PesdQL™3.0 Cancer Module, see Additional file 1. According to the original authors’ recommendations, the items were reverse-scored and linearly transformed to fit a 0–100 scale with higher scores indicating better HRQoL. Subscales and total scores were computed by taking the average of the item scores.

#### HRQoL (Chronic health conditions)

The Portuguese version of the short-form of the DISABKIDS Chronic Generic Measure [DCGM-12, 21] was used to assess HRQoL. This instrument assesses the HRQoL of children/adolescents aged 8–16 years old with chronic health conditions. According to recent recommendations [22], the final two items that concern medication were not included. The participants answered items (e.g., “Does your condition get you down?”, “Is your life ruled by your condition?”) on a 5-point Likert scale ranging from 1 (*never*) to 5 (*always*). After reverse-scoring six items, an overall score was obtained by averaging the sum of all items, with higher scores indicating better HRQoL. Cronbach’s alpha for the short-form was *r* = .84 [22]. For our sample, Cronbach’s alpha ranged from .78 (children) to .85 (adolescents).

#### Anxiety

Anxiety was assessed using the Portuguese version of the Revised Children’s Manifest Anxiety Scale - Second Edition (RCMAS-2). The RCMAS-2 is a self-report instrument designed to measure anxiety in children/adolescents between 6 and 19 years old [23]. The participants answered 10 items in short-form (e.g., “I often worry about something bad happening to me”, “I have too many headaches”) using a dichotomous response format. Higher scores indicate more anxiety. As reported in the technical manual, a test-retest reliability of the short-form over one-week was .54 and Cronbach’s alpha was .82 [23]. The Kuder-Richardson coefficient (KR-20) in the present sample was .68 for children and .62 for adolescents.

#### Intensity of treatment

The Portuguese version of the Intensity of Treatment Rating Scale 3.0 [ITR-3.0; 24] was used to assess the intensity of treatment. Fourteen pediatric oncologists used data from the medical records, blind to patient identity, to classify each child’s treatment into 1 of 4-levels of intensity, from 1 (*least intensive treatment*) to 4 (*most intensive treatment*), based on the diagnosis, the phase of illness (primary diagnosis or relapse), the stage/risk level for the patient, and the treatment modalities. Inter-rater reliability for a subset of this sample (*n* = 59) was k = .97 [25].

#### Socioeconomic status

The socioeconomic status of each family was classified in three levels (low, medium and high) using data from both parents’ job and educational level, according to an accepted classification system for the Portuguese context [26].

### Data analysis

Data analysis was conducted using Statistical Package for the Social Sciences (SPSS) and Analysis of Moment Structures (AMOS), versions 20.0 (IBM, SPSS Inc., Chicago, IL). Chi-square analyses were conducted to compare the two age groups regarding sociodemographic and clinical characteristics. Acceptance of the PedsQL™ 3.0 Cancer Module was assessed, by referring to the percentage of missing values per item. For descriptive and item analyses, the means, standard deviations, medians, interquartile ranges of the total score, subscales and items were computed. Floor and ceiling effects, skewness and kurtosis in the distributions of scores were also calculated. Floor or ceiling effects were considered to be present if more than 15 % of respondents achieved the lowest or highest possible score, respectively [27, 28]. Considering a sample size of more than 300 participants (*N* = 332), absolute skewness values of over 2 or absolute kurtosis over 7 were used as reference values for determining substantial non-normality [29, 30]. In addition, corrected item to total scale correlations were calculated; values *r* > .20 and ≥ .30 indicated moderate discrimination and good discrimination, respectively [31].

A CFA using robust Maximum likelihood (ML) was conducted to test whether the empirical data confirmed the factorial structure of the PedsQL™ 3.0 Cancer Module proposed in the theoretical model advanced by Varni et al. [13], which included a general second-order factor that informed eight specific subscales. A threshold of .40 was used for factor loadings [32]. To assess for model fit, evaluation of the chi-square statistic is recommended. However, because chi-square is sensitive to sample size [33], other goodness of fit indices were considered, including the comparative fit index (CFI) and the root mean square error of approximation (RMSEA) [12]. Values equal or above .90, and .95 for the CFI are considered acceptable and very good fit, respectively [34]. For RMSEA, a value lower than .01 indicates a great fit, values from .02 to .05 indicate a good fit and values up to .08 indicate an acceptable fit [34].

The convergent validity was assessed through Pearson correlation coefficients between the PedsQL™ 3.0 Cancer Module and the DCGM-12, an additional measure of HRQoL, and between the PedsQL™ 3.0 Cancer Module and the RCMAS-2, a measure of a different construct, that is, anxiety, which is theoretically related with HRQoL [35, 36].

The internal consistency was measured with Cronbach’s alpha coefficients [37] values were considered to be unacceptable (< .60), undesirable (.60–.65), minimally acceptable (.65–.70), respectable (.70–.80), and very good (.80–.90), according to DeVellis [35]. Test-retest reliability was assessed with intraclass correlation coefficient (ICC); values ≤ .40 were considered to be weak; values .41–.60 were considered to be moderate; values .61–.80 were considered to be good and values ≥ .81were considered to be excellent [38].

The effect sizes [39] are reported for comparison and association analyses (small: *r* ≥ .10, Cramer’s V ≥ .01; medium: *r* ≥ .30, Cramer’s V ≥ .03; large: *r* ≥ .50, Cramer’s V ≥ .05).

## Results

### Descriptive and item analyses

The percentage of missing items was 0 %. The means, standard deviations, medians, interquartile ranges, floor and ceiling effects, skewness, kurtosis, and corrected item to total scale correlations for the whole sample are presented in Table 2. There were no floor effects for the total score, subscales, and items, except for two items of the Worry subscale (W15, W16). There were ceiling effects for all items, six subscales (Pain and Hurt, Nausea, Procedural Anxiety, Treatment Anxiety, Perceived Physical Appearance, Communication) but not for the total score. The skewness statistic was −0.45 for the total score and ranged from −1.81 to −0.11 for the subscales and between −2.45 and 0.08 for the items. The kurtosis statistic was −0.17 for the total score and ranged from −0.99 to 2.69 for the subscales and between 1.33 and 6.14 for the items. Except for one item of the Treatment Anxiety subscale (TA11), skewness and kurtosis values of all items, all subscales and total score were below the thresholds of 2 and 7, respectively suggested normality was not violated. The item-total correlation coefficients (*r*s = .30 to .54) suggested good discrimination of the items.

**Table 2:** Descriptive and Item Analyses of the PedsQL™ 3.0 Cancer Module (N = 332)

Total Score / Subscales / Items	Mean	SD	Median	IQR	Floor, %	Ceiling, %	Skewness	Kurtosis	Corrected item-total correlations
Total Score	75.1	13.6	75.9	18.3	0.0	0.6	−0.45	−0.17	---
Pain and Hurt	81.0	21.8	87.5	37.5	0.6	42.8	−1.10	0.65	---
PH1	78.4	25.5	100.0	50.0	0.9	50.3	−0.86	−0.32	.44
PH2	83.7	23.3	100.0	25.0	1.8	59.3	−1.42	1.64	.40
Nausea	74.1	25.0	80.0	40.0	0.6	24.1	−0.81	−0.32	---
N3	70.5	34.6	100.0	50.0	9.3	50.3	−0.75	−0.79	.44
N4	72.8	32.2	87.5	50.0	7.2	50.0	−0.85	−0.43	.43
N5	81.9	28.0	100.0	44.0	3.9	64.5	−1.41	1.01	.46
N6	78.8	28.5	100.0	50.0	3.9	56.3	−1.16	0.38	.49
N7	66.4	32.1	75.0	50.0	7.2	38.3	−0.48	−0.87	.51
Procedural Anxiety	76.9	25.2	83.3	41.7	1.2	31.3	−1.09	0.36	---
PA8	69.1	31.1	75.0	50.0	8.1	36.4	−0.80	−0.31	.39
PA9	84.9	25.7	100.0	25.0	2.7	67.8	−1.69	2.04	.40
PA10	76.7	30.6	100.0	50.0	6.3	53.6	−1.16	0.29	.36
Treatment Anxiety	89.6	17.2	100.0	16.7	0.0	61.1	−1.81	2.69	---
TA11	90.7	19.7	100.0	0.0	1.2	76.8	−2.45	6.14	.39
TA12	91.3	17.6	100.0	0.0	0.0	76.8	−1.97	2.95	.51
TA13	86.8	23.3	100.0	25.0	2.1	69.3	−1.86	3.03	.51
Worry	55.6	28.4	58.3	50.0	3.0	10.2	−0.11	−0.99	---
W14	66.6	31.1	75.0	50.0	6.3	35.8	−0.49	−0.80	.54
W15	50.2	36.9	50.0	75.0	21.7	26.2	0.07	−1.33	.41
W16	49.9	34.8	50.0	50.0	18.4	21.7	0.08	−1.19	.48
Cognitive Problems	72.3	18.8	70.0	30.0	0.0	10.8	−0.43	−0.18	---
CP17	72.8	27.4	75.0	50.0	4.2	39.2	−0.79	0.03	.50
CP18	63.6	32.6	75.0	50.0	9.6	32.8	−0.45	−0.85	.32
CP19	74.5	27.0	75.0	50.0	4.5	41.0	−0.95	0.44	.39
CP20	72.1	27.6	75.0	50.0	3.9	38.0	−0.74	−0.15	.37
CP21	78.5	24.1	75.0	50.0	1.2	46.4	−0.89	0.11	.39
Perceived Physical Appearance	77.7	23.4	83.3	33.3	0.6	33.1	−0.97	0.20	---
PPA22	79.7	26.5	100.0	50.0	2.7	55.4	−1.11	0.43	.36
PPA23	77.5	33.8	100.0	44.0	9.6	60.8	−1.29	0.26	.30
PPA24	76.0	30.7	100.0	50.0	5.7	52.7	−1.07	0.02	.40
Communication	78.6	21.0	83.3	33.3	0.6	30.1	−0.97	0.56	---
C25	80.9	24.7	100.0	25.0	1.8	53.6	−1.20	0.81	.37
C26	80.7	24.5	100.0	25.0	1.8	52.7	−1.19	0.83	.42
C27	74.2	30.2	75.0	50.0	5.4	47.0	−0.95	−0.11	.31

*Note.* *SD* standard deviation; *IQR* interquartile range

### Validity

#### Factorial validity

**Fig. 1:**
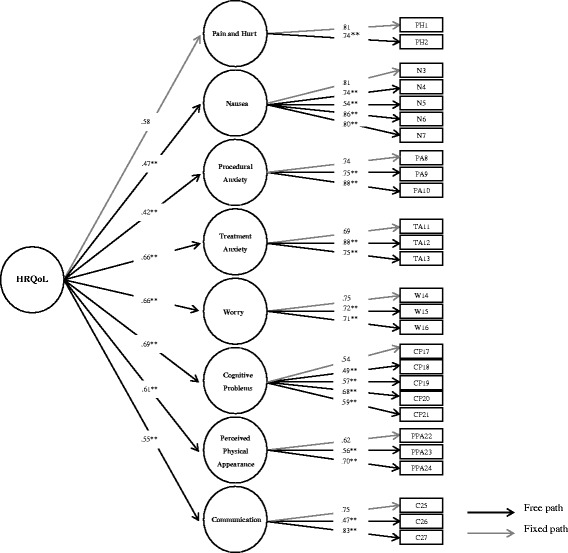
Fit indexes for the model: χ^2^_316_ = 590.80; *p* < .001; CFI = .91; RMSEA = .05. Values shown in the figure represent completely standardized regression weights of the factor loadings. **p < .01

Results are provided in Fig. 1. A second-order CFA, which comprised eight first-order factors and one second-order factor, demonstrated acceptable fit to the data (χ^2^_316_ = 590.80; *p* < .001; CFI = .91; RMSEA = .05, 90 % CI .05, .06). All standardized items loaded significantly in their respective factors, with the factor loadings ranging from .47 to .88. Similarly, all eight first-order factors loaded significantly in the second-order factor, with the factor loadings ranging from .42 to .69.

#### Convergent validity

As shown in Table 3, the PedsQL™ 3.0 Cancer Module was significantly and positively correlated with DCGM-12 and negatively correlated with the RCMAS-2, with the exception of the children’s reports of Nausea and Procedural Anxiety, which did not reach significance. For the whole sample and both age groups, most correlations were medium to large.

**Table 3:** Convergent Validity of the PedsQL™ 3.0 Cancer Module

	DCGM-12^a^			RCMAS-2^a^		
	Whole sample	Children	Adolesc	Whole sample	Children	Adolesc
	(*N* = 332)	(*n* = 143)	(*n* = 189)	(*N* = 332)	(*n* = 143)	(*n* = 189)
Total score	.58**	.61**	.56**	-.51**	-.46**	-.56**
Pain and Hurt	.46**	.34**	.55**	-.33**	-.26**	-.40**
Nausea	.43**	.42**	.44**	-.16**	-.14	-.18**
Procedural Anxiety	.17**	.21**	.15*	-.17**	-.09	-.26**
Treatment Anxiety	.29**	.35**	.26**	-.29**	-.26**	-.33**
Worry	.34**	.37**	.34**	-.32**	-.36**	-.33**
Cognitive Problems	.39**	.43**	.36**	-.44**	-.40**	-.48**
Perceived Physical Appearance	.35**	.33**	.37**	-.45**	-.40**	-.52**
Communication	.32**	.37**	.28**	-.37**	-.31**	-.43**

*Note.* Adolesc = adolescents

**p ≤ .01. *p < .05

^a^Pearson correlations coefficients

### Reliability

As indicated in Table 4, for the whole sample and both age groups, the total scale had very good internal consistency. For the subscales, the scores were more heterogeneous, with values that ranged from minimally acceptable to very good internal consistency, with the exception of the children’s reports of Pain and Hurt, Cognitive Problems, and Perceived Physical Appearance. With regard to the test-retest ICC values for the total scale and subscales, these were good to excellent, with the exception of Pain and Hurt and Cognitive Problems for children, who presented moderate values (Table 4).

**Table 4:** Internal Consistency and Test-retest Reliability of the PedsQL™ 3.0 Cancer Module

	Internal consistency^a^			Test-retest reliability (one-weak)^b^		
	Whole sample	Children	Adolesc	Whole sample	Children	Adolesc
	(*N* = 332)	(*n* = 143)	(*n* = 189)	(*N* = 52)	(*n* = 22)	(*n* = 30)
Total score	.87	.83	.89	.79**	.70**	.83**
Pain and Hurt	.75	.58	.85	.61**	.54**	.63**
Nausea	.86	.79	.90	.78**	.78**	.78**
Procedural Anxiety	.82	.78	.87	.81**	.76**	.86**
Treatment Anxiety	.80	.68	.87	.72**	.89**	.63**
Worry	.77	.69	.81	.76**	.68**	.79**
Cognitive Problems	.70	.61	.77	.77**	.48**	.87**
Perceived Physical Appearance	.65	.49	.75	.81**	.85**	.78**
Communication	.70	.69	.70	.74**	.74**	.75**

*Note.* Adolesc = adolescents

**p ≤ .01

^a^Cronbach’s alpha; ^b^Intraclass correlation coefficients

## Discussion

The aim of this study was to assess the psychometric properties of the European Portuguese version of the PedsQL™ 3.0 Cancer Module for children and adolescents. The findings supported the factorial structure and the convergent validity. Overall, results also suggested good internal consistency and temporal stability.

The absence of missing data suggested that children and adolescents were willing and able to provide data. Similar to other versions [e.g. 14–16, 20] there was a tendency for ceiling effect for the subscales and items. This tendency has been reported in other studies concerning the measurement of HRQoL for children/adolescents with cancer [40]. The inclusion of children/adolescents that were off-treatment may have potentially accentuated the ceiling effects [20]. This study increases current knowledge by showing that total scores and all subscales of the PedsQL 3.0 Cancer Module were normally distributed, a trend that has been reported in other studies [14, 15]. In addition, similar to the Brazilian version [17], item-total correlations demonstrated good item discrimination.

Although the PedsQL™ 3.0 Cancer Module has been translated into 25 languages and some psychometric properties of these versions have been reported [e.g., 14, 15, 16], to our knowledge, this is the first study to analyze the factorial structure of this instrument via CFA. In accordance with the factorial structure proposed by the original authors [13], the CFA indicated a second-order factorial model and determined that the eight first-order factors are structurally related and consistent indicators of a higher level construct, that is, the HRQoL.

The correlations between the PedsQL™ 3.0 Cancer Module and the DCGM-12, and the RCMAS-2 scores attested the convergent validity. The medium to large correlations between the two instruments, that measure the HRQoL, were similar compared to the original version [13], the Japanese version [15] and the Chinese version [14] that compared the PedsQL™ 3.0 Cancer Module and the PedsQL™ 4.0 Generic Core Scales. The unique contributions of our study, compared with previous research with the PedsQL™ 3.0 Cancer Module [13–17], are that the data are presented separately for each age group and that the other scale that assessed the HRQoL (DCGM-12) was not derived from the same theoretical framework. In addition, the majority of the correlations between the PedsQL™ 3.0 Cancer Module and the RCMAS-2 scores were also medium to large, with the exception of the children’s reports of Nausea and Procedural Anxiety. However, the items of the RCMAS-2 are more oriented for physiological anxiety, worry, and social anxiety [23] and not for the physical side effects of cancer treatment (e.g., nausea, dysgeusia) or for the anxiety related to procedures (e.g., injections, blood tests, intravenous therapy).

With regard to reliability, PedsQL™ 3.0 Cancer Module exceeded the minimally acceptable internal consistency cut-point, with the exception of the Pain and Hurt, Cognitive Problems, and Perceived Physical Appearance subscales in the self-reports of children. Similar to these findings, others studies have also demonstrated poor internal consistency for one or all aforementioned subscales [13, 17]. The low reliability of these subscales identified across countries may be related to their small number of items [41] and also to children’s learning difficulties, which may mirror the cognitive and neuropsychological effects of the treatment and the high rate of absenteeism [42]. Although, in Portugal, the education of youth with cancer is protected by Law (No. 71/2009 of August 6^th^) [43] with school support being available upon request at home and at the hospital, the treatment for pediatric cancer can often interrupt regular school attendance, that might be more critical for the children's group.

Finally, total score and all subscales demonstrated good to excellent test-retest reliability, with the exception of two subscales for children. Similar to Lau et al. [14], one of the only two studies presenting the test-retest reliability for this age group, the subscales Pain and Hurt and Cognitive Problems (for children) presented moderate test-retest reliability values. However, the children/adolescents who participated in the test-retest were undergoing treatment at the time of the study. Thus, the low values in the aforementioned subscales may reflect changes over the previous week (e.g., treatment side effects, outpatient or inpatient treatment, interrupt homebound services) or impact due to a specific situation (e.g., blood test, lumbar punctures) [44]. With few exceptions, however, the overall results suggest that the Portuguese version of the PedsQL™ 3.0 Cancer Module is stable over time.

The strengths of the PedsQL™ 3.0 Cancer Module include the sound theoretical background, the inclusion of the child-centered features, that is, important domains of a child’s life, which enables the child to assess his/her own HRQoL taking into account his/her developmental stage [45] according to the guidelines in this field [46, for a review]. A strength of this specific study is its large sample size, which allowed for analyzing the convergent validity and reliability of this measure separately for children and adolescents. Additionally, this was the first study to conduct the factorial validity of the PedsQL™ 3.0 Cancer Module via CFA, providing empirical support for the original model proposed by the original authors of this scale.

The current study also presents potential limitations. First, the sample collection was conducted in three of the four-oncology centers in Portugal. Although it is unlikely that regional differences may influence how children/adolescents perceive the impact of cancer on their own HRQoL, generalizations should be made with caution. Second, we did not control for the treatments and their side effects, and school support over the two assessment moments. Third, an analysis of the parents’ proxy-reports was not conducted. Future research should inspect the psychometric properties of parents’ proxy-reports and the agreement between the parents’ proxy-reports and the children/adolescents’ self-reports. Moreover, future studies with a larger sample should analyze whether the factorial structure remains valid across groups of children and adolescents and across on- and off-treatment status.

## Conclusions

This approach enlarged the potential use of the PedsQL™ 3.0 Cancer Module in future research by demonstrating the validity of assessing both a general HRQoL factor and the eight specific subscales provided by each subscale. The study established that the European Portuguese version of the PedsQL™3.0 Cancer Module for child and adolescent self-report was a reliable and valid measurement instrument to assess children/adolescent reports of HRQoL, thereby expanding the potential for cross-cultural applications of the PedsQL™ 3.0 Cancer Module.

## Additional file

Additional file 1:
**The Portuguese and the English originals versions of the PedsQL™ 3.0 Cancer Module: Child report (ages 8-12) and Teen report (ages 13-18).** (PDF 1029 kb)
