# Supplementary material for: Psychometric study of the European Portuguese version of the PedsQL 3.0 Cancer Module
Source: Health Qual Life Outcomes. 2016 Feb 13;14:20. doi: 10.1186/s12955-016-0421-y (PMC4752803; doi:10.1186/s12955-016-0421-y)
Supplement: Additional file 1: — The Portuguese and the English originals versions of the PedsQL™ 3.0 Cancer Module: Child report (ages 8-12) and Teen report (ages 13-18). (PDF 1029 kb) [file 12955_2016_421_MOESM1_ESM.pdf]

Additional file 1
The Portuguese and the English originals versions of the PedsQL™ 3.0 Cancer Module:
Child report (ages 8-12) and Teen report (ages 13-18)

PedsQL™

Módulo do Cancro

Versão 3.0

Relato da Criança (8-12 anos)

Review Copy
Do not use without permission

INSTRUÇÕES

Por vezes, crianças com cancro têm alguns problemas. Por favor, diz-nos **até que ponto** cada uma destas situações tem sido um problema para ti durante o **último mês.** Por favor, assinala a tua resposta com um círculo à volta de uma das opções:

0 se **nunca** é um problema
1 se **quase nunca** é um problema
2 se **algumas vezes** é um problema
3 se **muitas vezes** é um problema
4 se **quase sempre** é um problema

Não há respostas certas ou erradas.
Se não entenderes uma pergunta, por favor pede ajuda.

Durante o **último mês**, até que ponto tem sido um problema para ti

**DOR** (problemas com...)		Nunca	Quase nunca	Algumas vezes	Muitas vezes	Quase sempre
1	Tenho dores nas articulações e/ou músculos.	0	1	2	3	4
2	Tenho muitas dores.	0	1	2	3	4

**NÁUSEA** (problemas com...)		Nunca	Quase nunca	Algumas vezes	Muitas vezes	Quase sempre
1	Fico enjoado quando tenho tratamentos médicos.	0	1	2	3	4
2	A comida não me sabe muito bem.	0	1	2	3	4
3	Fico enjoado quando penso nos tratamentos médicos.	0	1	2	3	4
4	Sinto-me demasiado enjoado para comer.	0	1	2	3	4
5	Algumas comidas e cheiros enjoam-me.	0	1	2	3	4

**ANSIEDADE NOS PROCEDIMENTOS** (problemas com...)		Nunca	Quase nunca	Algumas vezes	Muitas vezes	Quase sempre
1	As picadas (ex: injeções, colheitas de sangue, endovenosas) doem-me.	0	1	2	3	4
2	Fico com medo quando tenho de tirar sangue.	0	1	2	3	4
3	Fico com medo quando tenho de ser picado (ex: injeções, colheitas de sangue, endovenosas).	0	1	2	3	4

**ANSIEDADE NOS TRATAMENTOS** (problemas com...)		Nunca	Quase nunca	Algumas vezes	Muitas vezes	Quase sempre
1	Fico com medo quando estou à espera do médico.	0	1	2	3	4
2	Fico com medo quando tenho de ir ao médico.	0	1	2	3	4
3	Fico com medo quando tenho de ir ao hospital.	0	1	2	3	4

**PREOCUPAÇÃO** (problemas com...)		Nunca	Quase nunca	Algumas vezes	Muitas vezes	Quase sempre
1	Preocupo-me com os efeitos secundários dos tratamentos médicos.	0	1	2	3	4
2	Preocupo-me se os tratamentos médicos estão ou não a resultar.	0	1	2	3	4
3	Preocupo-me se o cancro vai voltar ou recidivar.	0	1	2	3	4

**PROBLEMAS COGNITIVOS** (problemas com...)		Nunca	Quase nunca	Algumas vezes	Muitas vezes	Quase sempre
1	Tenho dificuldade em saber o que fazer quando alguma coisa me incomoda.	0	1	2	3	4
2	Tenho dificuldade em resolver problemas de matemática.	0	1	2	3	4
3	Tenho dificuldade em fazer trabalhos escritos ou relatórios para a escola.	0	1	2	3	4
4	Tenho dificuldade em prestar atenção às coisas.	0	1	2	3	4
5	Tenho dificuldade em lembrar-me do que leio.	0	1	2	3	4

Review Copy
Do not use without permission

Durante o **último mês**, até que ponto tem sido um problema para ti

	**PERCEÇÃO DA APARÊNCIA FÍSICA** (problemas com...)	**Nunca**	**Quase nunca**	**Algumas vezes**	**Muitas vezes**	**Quase sempre**
**1**	Sinto que não sou bonito.	**0**	**1**	**2**	**3**	**4**
**2**	Não gosto que outras pessoas vejam as minhas cicatrizes.	**0**	**1**	**2**	**3**	**4**
**3**	Fico envergonhado quando outras pessoas veem o meu corpo.	**0**	**1**	**2**	**3**	**4**

	**COMUNICAÇÃO** (problemas com...)	**Nunca**	**Quase nunca**	**Algumas vezes**	**Muitas vezes**	**Quase sempre**
**1**	Tenho dificuldade em dizer aos médicos e enfermeiros o que sinto.	**0**	**1**	**2**	**3**	**4**
**2**	Tenho dificuldade em fazer perguntas aos médicos e enfermeiros.	**0**	**1**	**2**	**3**	**4**
**3**	Tenho dificuldade em explicar a minha doença a outras pessoas.	**0**	**1**	**2**	**3**	**4**

Review Copy
Do not use without permission

PedsQL™

Módulo do Cancro

Versão 3.0

Relato do Jovem (13-18 anos)

Review Copy
Do not use without permission

INSTRUÇÕES

Por vezes, crianças/jovens com cancro têm alguns problemas. Por favor, diz-nos **até que ponto** cada uma destas situações tem sido um problema para ti durante o **último mês.** Por favor, assinala a tua resposta com um círculo à volta de uma das opções:

0 se **nunca** é um problema
1 se **quase nunca** é um problema
2 se **algumas vezes** é um problema
3 se **muitas vezes** é um problema
4 se **quase sempre** é um problema

Não há respostas certas ou erradas.
Se não entenderes uma pergunta, por favor pede ajuda.

Durante o **último mês**, até que ponto tem sido um problema para ti

**DOR** (problemas com...)		Nunca	Quase nunca	Algumas vezes	Muitas vezes	Quase sempre
**1**	Tenho dores nas articulações e/ou músculos.	0	1	2	3	4
**2**	Tenho muitas dores.	0	1	2	3	4

**NÁUSEA** (problemas com...)		Nunca	Quase nunca	Algumas vezes	Muitas vezes	Quase sempre
**1**	Fico enjoado quando tenho tratamentos médicos.	0	1	2	3	4
**2**	A comida não me sabe muito bem.	0	1	2	3	4
**3**	Fico enjoado quando penso nos tratamentos médicos.	0	1	2	3	4
**4**	Sinto-me demasiado enjoado para comer.	0	1	2	3	4
**5**	Algumas comidas e cheiros enjoam-me.	0	1	2	3	4

**ANSIEDADE NOS PROCEDIMENTOS** (problemas com...)		Nunca	Quase nunca	Algumas vezes	Muitas vezes	Quase sempre
**1**	As picadas (ex: injeções, colheitas de sangue, endovenosas) doem-me.	0	1	2	3	4
**2**	Fico com medo quando tenho de tirar sangue.	0	1	2	3	4
**3**	Fico com medo quando tenho de ser picado (ex: injeções, colheitas de sangue, endovenosas).	0	1	2	3	4

**ANSIEDADE NOS TRATAMENTOS** (problemas com...)		Nunca	Quase nunca	Algumas vezes	Muitas vezes	Quase sempre
**1**	Fico com medo quando estou à espera do médico.	0	1	2	3	4
**2**	Fico com medo quando tenho de ir ao médico.	0	1	2	3	4
**3**	Fico com medo quando tenho de ir ao hospital.	0	1	2	3	4

**PREOCUPAÇÃO** (problemas com...)		Nunca	Quase nunca	Algumas vezes	Muitas vezes	Quase sempre
**1**	Preocupo-me com os efeitos secundários dos tratamentos médicos.	0	1	2	3	4
**2**	Preocupo-me se os tratamentos médicos estão ou não a resultar.	0	1	2	3	4
**3**	Preocupo-me se o cancro vai voltar ou recidivar.	0	1	2	3	4

**PROBLEMAS COGNITIVOS** (problemas com...)		Nunca	Quase nunca	Algumas vezes	Muitas vezes	Quase sempre
**1**	Tenho dificuldade em saber o que fazer quando alguma coisa me incomoda.	0	1	2	3	4
**2**	Tenho dificuldade em resolver problemas de matemática.	0	1	2	3	4
**3**	Tenho dificuldade em fazer trabalhos escritos ou relatórios para a escola.	0	1	2	3	4
**4**	Tenho dificuldade em prestar atenção às coisas.	0	1	2	3	4
**5**	Tenho dificuldade em lembrar-me do que leio.	0	1	2	3	4

Review Copy
Do not use without permission

Durante o **último mês**, até que ponto tem sido um problema para ti

**PERCEÇÃO DA APARÊNCIA FÍSICA** (problemas com...)		**Nunca**	**Quase nunca**	**Algumas vezes**	**Muitas vezes**	**Quase sempre**
**1**	Sinto que não sou bonito.	**0**	**1**	**2**	**3**	**4**
**2**	Não gosto que outras pessoas vejam as minhas cicatrizes.	**0**	**1**	**2**	**3**	**4**
**3**	Fico envergonhado quando outras pessoas veem o meu corpo.	**0**	**1**	**2**	**3**	**4**

**COMUNICAÇÃO** (problemas com...)		**Nunca**	**Quase nunca**	**Algumas vezes**	**Muitas vezes**	**Quase sempre**
**1**	Tenho dificuldade em dizer aos médicos e enfermeiros o que sinto.	**0**	**1**	**2**	**3**	**4**
**2**	Tenho dificuldade em fazer perguntas aos médicos e enfermeiros.	**0**	**1**	**2**	**3**	**4**
**3**	Tenho dificuldade em explicar a minha doença a outras pessoas.	**0**	**1**	**2**	**3**	**4**

Review Copy
Do not use without permission

ID# ___________________________

Date:___________________________

PedsQL ™

Cancer Module

Version 3.0

CHILD REPORT (ages **8-12**)

Review copy
Do not use without permission

DIRECTIONS

Children with cancer sometimes have special problems. Please tell us **how much of a problem** each one has been for you during the **past one month** by circling:

0 if it is **never** a problem
1 if it is **almost never** a problem
2 if it is **sometimes** a problem
3 if it is **often** a problem
4 if it is **almost always** a problem

There are no right or wrong answers.
If you do not understand a question, please ask for help.

Not to be reproduced without permission

Copyright © 1998 JW Varni, Ph.D.
All rights reserved

*In the past **one month,** how much of a **problem** has this been for you*

**PAIN AND HURT** ***(problems with)***	**Never**	**Almost Never**	**Some-times**	**Often**	**Almost Always**
1. I ache or hurt in my joints and/or muscles	0	1	2	3	4
2. I hurt a lot	0	1	2	3	4

**NAUSEA** ***(problems with)***	**Never**	**Almost Never**	**Some-times**	**Often**	**Almost Always**
1. I become sick to my stomach when I have medical treatments	0	1	2	3	4
2. Food does not taste very good to me	0	1	2	3	4
3. I become sick to my stomach when I think about medical treatments	0	1	2	3	4
4. I feel too sick to my stomach to eat	0	1	2	3	4
5. Some foods and smells make me sick to my stomach	0	1	2	3	4

**PROCEDURAL ANXIETY** ***(problems with)***	**Never**	**Almost Never**	**Some-times**	**Often**	**Almost Always**
1. Needle sticks (i.e. injections, blood tests, IV's) hurt	0	1	2	3	4
2. I get scared when I have to have blood tests	0	1	2	3	4
3. I get scared about having needle sticks (i.e. injections, blood tests, IV's)	0	1	2	3	4

**TREATMENT ANXIETY** ***(problems with)***	**Never**	**Almost Never**	**Some-times**	**Often**	**Almost Always**
1. I get scared when I am waiting to see the doctor	0	1	2	3	4
2. I get scared when I have to go to the doctor	0	1	2	3	4
3. I get scared when I have to go to the hospital	0	1	2	3	4

**WORRY** ***(problems with)***	**Never**	**Almost Never**	**Some-times**	**Often**	**Almost Always**
1. I worry about side effects from medical treatments	0	1	2	3	4
2. I worry about whether or not my medical treatments are working	0	1	2	3	4
3. I worry that my cancer will come back or relapse	0	1	2	3	4

**COGNITIVE PROBLEMS** ***(problems with)***	**Never**	**Almost Never**	**Some-times**	**Often**	**Almost Always**
1. It is hard for me to figure out what to do when something bothers me	0	1	2	3	4
2. I have trouble solving math problems	0	1	2	3	4
3. I have trouble writing school papers or reports	0	1	2	3	4
4. It is hard for me to pay attention to things	0	1	2	3	4
5. It is hard for me to remember what I read	0	1	2	3	4

Do not use without permission
Review copy

*In the past **one month,** how much of a **problem** has this been for you*

Not to be reproduced without permission
Copyright © 1998 JW Varni, Ph.D.
All rights reserved

**PERCEIVED PHYSICAL APPEARANCE** ***(problems with)***	**Never**	**Almost Never**	**Some-times**	**Often**	**Almost Always**
1. I feel I am not good looking	0	1	2	3	4
2. I don't like other people to see my scars	0	1	2	3	4
3. I am embarrassed when others see my body	0	1	2	3	4

**COMMUNICATION** ***(problems with)***	**Never**	**Almost Never**	**Some-times**	**Often**	**Almost Always**
1. It is hard for me to tell the doctors and nurses how I feel	0	1	2	3	4
2. It is hard for me to ask the doctors and nurses questions	0	1	2	3	4
3. It is hard for me to explain my illness to other people	0	1	2	3	4

Review copy
Do not use without permission

Not to be reproduced without permission
Copyright © 1998 JW Varni, Ph.D.
All rights reserved

ID# ___________________________

Date:_________________________

PedsQL™

Cancer Module

Version 3.0

TEEN REPORT (ages **13-18**)

Review copy
Do not use without permission

DIRECTIONS

Teens with cancer sometimes have special problems. Please tell us **how much of a problem** each one has been for you during the **past one month** by circling:

0 if it is **never** a problem
1 if it is **almost never** a problem
2 if it is **sometimes** a problem
3 if it is **often** a problem
4 if it is **almost always** a problem

There are no right or wrong answers.
If you do not understand a question, please ask for help.

*In the past **one month,** how much of a **problem** has this been for you …*

Not to be reproduced without permission
Copyright © 1998 JW Varni, Ph.D.
All rights reserved

**PAIN AND HURT** ***(problems with…)***	**Never**	**Almost Never**	**Some-times**	**Often**	**Almost Always**
1. I ache or hurt in my joints and/or muscles	0	1	2	3	4
2. I hurt a lot	0	1	2	3	4

**NAUSEA** ***(problems with…)***	**Never**	**Almost Never**	**Some-times**	**Often**	**Almost Always**
1. I become sick to my stomach when I have medical treatments	0	1	2	3	4
2. Food does not taste very good to me	0	1	2	3	4
3. I become sick to my stomach when I think about medical treatments	0	1	2	3	4
4. I feel too sick to my stomach to eat	0	1	2	3	4
5. Some foods and smells make me sick to my stomach	0	1	2	3	4

**PROCEDURAL ANXIETY** ***(problems with…)***	**Never**	**Almost Never**	**Some-times**	**Often**	**Almost Always**
1. Needle sticks (i.e. injections, blood tests, IV's) hurt	0	1	2	3	4
2. I get scared when I have to have blood tests	0	1	2	3	4
3. I get scared about having needle sticks (i.e. injections, blood tests, IV's)	0	1	2	3	4

**TREATMENT ANXIETY** ***(problems with…)***	**Never**	**Almost Never**	**Some-times**	**Often**	**Almost Always**
1. I get scared when I am waiting to see the doctor	0	1	2	3	4
2. I get scared when I have to go to the doctor	0	1	2	3	4
3. I get scared when I have to go to the hospital	0	1	2	3	4

**WORRY** ***(problems with…)***	**Never**	**Almost Never**	**Some-times**	**Often**	**Almost Always**
1. I worry about side effects from medical treatments	0	1	2	3	4
2. I worry about whether or not my medical treatments are working	0	1	2	3	4
3. I worry that my cancer will come back or relapse	0	1	2	3	4

**COGNITIVE PROBLEMS** ***(problems with…)***	**Never**	**Almost Never**	**Some-times**	**Often**	**Almost Always**
1. It is hard for me to figure out what to do when something bothers me	0	1	2	3	4
2. I have trouble solving math problems	0	1	2	3	4
3. I have trouble writing school papers or reports	0	1	2	3	4
4. It is hard for me to pay attention to things	0	1	2	3	4
5. It is hard for me to remember what I read	0	1	2	3	4

*In the past **one month,** how much of a **problem** has this been for you …*

Review copy
Do not use without permission

Not to be reproduced without permission
Copyright © 1998 JW Varni, Ph.D.
All rights reserved

**PERCEIVED PHYSICAL APPEARANCE** ***(problems with…)***	**Never**	**Almost Never**	**Some-times**	**Often**	**Almost Always**
1. I feel I am not good looking	0	1	2	3	4
2. I don't like other people to see my scars	0	1	2	3	4
3. I am embarrassed when others see my body	0	1	2	3	4

**COMMUNICATION** ***(problems with…)***	**Never**	**Almost Never**	**Some-times**	**Often**	**Almost Always**
1. It is hard for me to tell the doctors and nurses how I feel	0	1	2	3	4
2. It is hard for me to ask the doctors and nurses questions	0	1	2	3	4
3. It is hard for me to explain my illness to other people	0	1	2	3	4

Review copy
Do not use without permission

Not to be reproduced without permission

Copyright © 1998 JW Varni, Ph.D.
All rights reserved
